# PSGL-1 is an evolutionarily conserved antiviral restriction factor

**DOI:** 10.1128/mbio.00387-23

**Published:** 2023-10-03

**Authors:** Chao Jiang, Miao Mei, Ying Liu, Min Hou, Jun Jiao, Ya Tan, Xu Tan

**Affiliations:** 1 Tsinghua-Peking Center for Life Sciences, MOE Key Laboratory of Bioorganic Phosphorus Chemistry & Chemical Biology, School of Pharmaceutical Sciences, Tsinghua University, Beijing, China; 2 Chinese Institutes for Medical Research, Beijing, China; 3 Global Health Institute, Swiss Federal Institute of Technology Lausanne (EPFL), Lausanne, Switzerland; University of Pittsburgh School of Medicine, Pittsburgh, Pennsylvania, USA; The Ohio State University, Columbus, Ohio, USA

**Keywords:** PSGL-1, HIV-1, murine leukemia virus, restriction factor

## Abstract

The arms race between viruses and their hosts shaped the evolutionary history and the genome composition of both parties. Restriction factors are the first-line antiviral effectors encoded by the host genomes and are often conserved through evolution to protect the hosts from morbidity, mortality, and even extinction associated with viral infections. There are a number of restriction factors identified so far to counteract HIV infection of the humans. PSGL-1 is a recently characterized human restriction factor that acts both early and late in the viral life cycle, the latter of which is antagonized by the HIV-1 Vpu protein. Here we show *in vitro* and in a knockout mouse model that PSGL-1’s antiviral function is conserved in mice for combating the murine leukemia virus (MLV). In counter-strike, the glycogag or glycoMA proteins encoded by MLV can interact with mouse PSGL-1, which leads to Cul3-KLHL20 E3 ligase-dependent ubiquitination and degradation of PSGL-1. The amino acids involved in this interaction demonstrate the evidence of positive selection, manifesting the evolution pressure from the antagonism between PSGL-1 and glycogag/glycoMA. Our data support that PSGL-1 is an evolutionarily conserved antiviral restriction factor.

**Importance:**

Studying the co-evolution between viruses and humans is important for understanding why we are what we are now as well as for developing future antiviral drugs. Here we pinned down an evolutionary arms race between retroviruses and mammalian hosts at the molecular level by identifying the antagonism between a host antiviral restriction factor PSGL-1 and viral accessory proteins. We show that this antagonism is conserved from mouse to human and from mouse retrovirus to HIV. Further studying this antagonism might provide opportunities for developing new antiviral therapies.

## INTRODUCTION

As illustrated by the red queen hypothesis, viruses and their hosts are undergoing a constant evolutionary arms race through genetic mutations and phenotypic variations to maintain the status quo of the antagonistic relationship (1). An outcome of this arms race is that the genes involved demonstrate a higher rate of mutations through evolution than other genes, which is termed positive selection on these genes (1, 2). This phenomenon is probably best studied with the so-called restriction factors encoded by the host genome that have potent antiviral activities, which are often antagonized by the viruses of the particular hosts (3). In the case of HIV, several restriction factors have been uncovered so far, including APOBEC3, tetherin, SAMHD1, SERINC5, etc. (4 – 8). Remarkably, these restriction factors are antagonized by HIV accessory proteins often via induced protein ubiquitination (9, 10). These studies deepened our understanding of the antiviral mechanisms of our innate immunity and provided new targets for developing antiviral drugs.

Recently, we have identified a new restriction factor named PSGL-1, which demonstrated potent inhibition of HIV infectivity but is partially antagonized by the HIV-1 Vpu protein through Vpu-induced ubiquitination and subsequent proteasomal degradation of PSGL-1 (11). Vpu recruits the cullin-RING E3 ligase (CRL) complex SCF^βTrCP2^ to promote K48 chain type polyubiquitination of PSGL-1, which is subsequently degraded by the proteasome (11). In a sequel study, we showed that the mechanisms of PSGL-1’s action might be due to its ability to exclude HIV envelope proteins from new viral particles as well as its function to restrict actin depolymerization to block HIV-1 reverse transcription (12). Others have data supporting a physical barrier formed by PSGL-1 on the surface of viral particles that prevents viral attachment to target cells (13, 14). We also demonstrated that PSGL-1 is an interferon gamma (IFN-γ) inducible gene and contributes to the antiviral activity of IFN-γ in activated human primary CD4+T cells (11). The following studies also uncovered PSGL-1’s antiviral activity to other viruses including murine leukemia virus (MLV), influenza virus, and SARS-CoV-2, showing the broad spectrum of the viral restriction function of PSGL-1 (13, 15). However, PSGL-1’s antiviral activity *in vivo* and whether this activity is conserved during evolution are yet to be examined. Here by using a mouse model of MLV infection, we demonstrate that PSGL-1 is a conserved IFN-γ inducible restriction factor for retrovirus in mice. We also provide evidence of the positive selection of PSGL-1 amino acid sequences in the virus-host arms race, suggesting the importance of this restriction factor through mammalian evolution.

## RESULTS

### Both human and mouse PSGL-1 inhibit MLV replication

**Fig 1:**
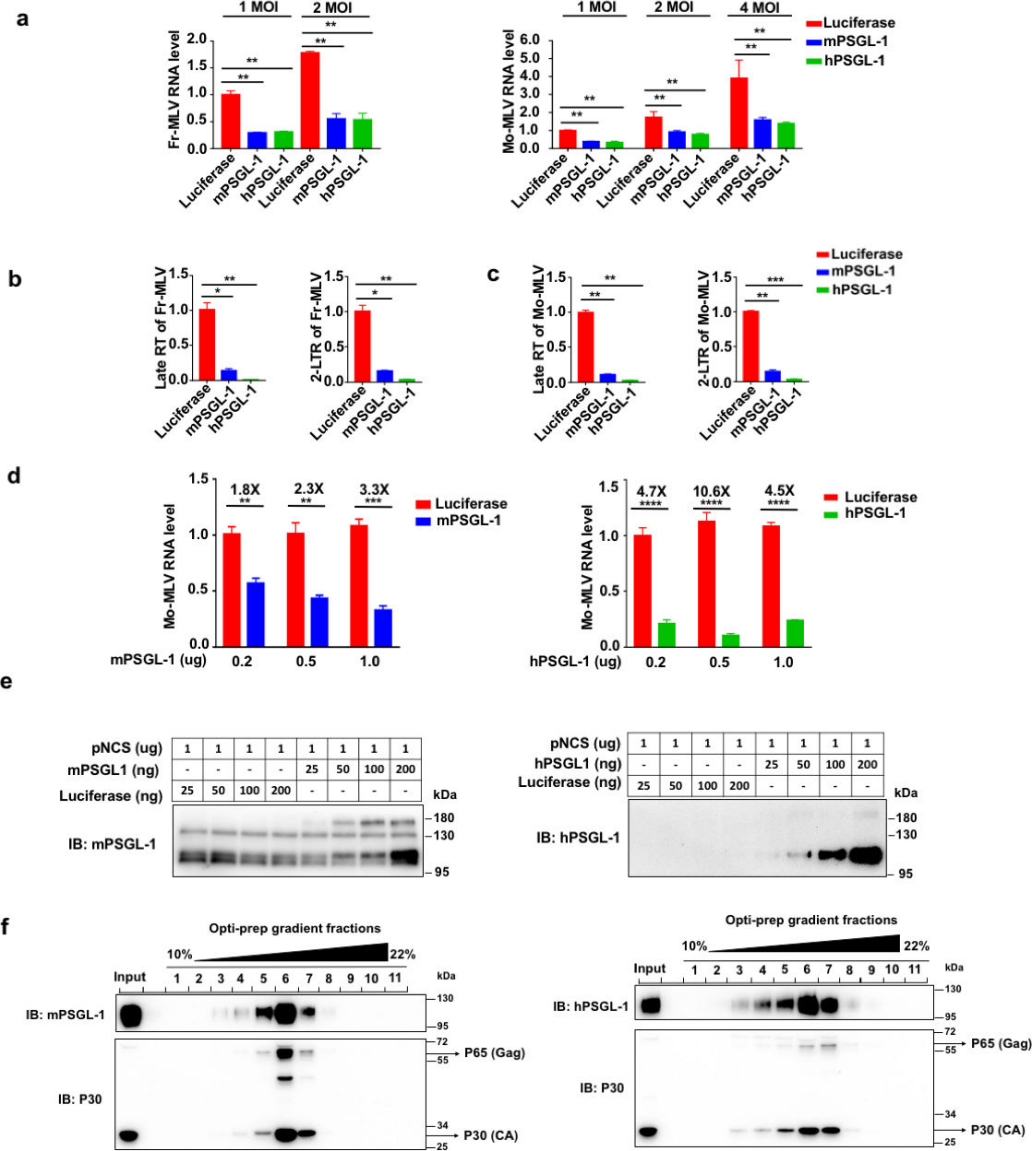
PSGL-1 inhibits MLV infection in early and late stages. (a) Murine 3T3 cells overexpressing m/hPSGL-1 were infected by Fr-MLV (left) or Mo-MLV (right) for 48 h with the increasing multiplicity of infection (MOI) as shown, then the RNA levels of viruses were detected by RT-qPCR. (b and c) 3T3 cells as in (a) were infected by Fr-MLV (b) or Mo-MLV (c) with 2 MOI for 24 h before the DNA was extracted to measure late reverse transcription (RT) products and 2-LTR (long terminal repeat) circles. (d) The RNA levels of Mo-MLV isolated from the supernatant of HEK293T cells co-transfected with the Mo-MLV packaging plasmid pNCS and mouse (left) or human (right) PSGL-1 expressing plasmid for 48 h were measured and normalized, then viruses of equal amounts of RNA were used to infect naive 3T3 cells for 48 h. The RNA levels of Mo-MLV in 3T3 cells were detected with RT-qPCR. (e) Western blots of supernatant of HEK293T cells as (d) to detect PSGL-1 within the released MLV particles. (f) 293T cells were co-transfected with pNCS and mouse or human PSGL-1 expressing vectors. Two days post-transfection, viral particles in the supernatants were pelleted and then subjected to density gradient centrifugation in 10% to 22% Opti-prep solution. Fractions were collected, with fraction 1 referring to the top of the gradient. Input: the unfractionated samples. The results of (a–f) are representative of at least three independent experiments.

We previously showed that overexpression of human PSGL-1 in a number of cell lines potently inhibited HIV-1 reverse transcription and also the infectivity of progeny virions (11, 12). We overexpressed both human and mouse PSGL-1 (shorthanded hereafter as hPSGL-1 and mPSGL-1, respectively) in murine fibroblast cell line 3T3 and found both PSGL-1’s demonstrated similar antiviral activities to two strains of MLV, Moloney MLV, and Friend MLV(16) (Fig. 1a; Fig. S1a). The DNA levels of the late RT products of the reverse transcription of two strains of MLV are significantly lower in 3T3 cells overexpressing PSGL-1 as measured using RT-qPCR, consistent with the reverse transcription inhibition observed in our previous studies (Fig. 1b and c). We also co-transfected the Mo-MLV plasmid pNCS and a PSGL-1 expressing plasmid into HEK293T cells, which then released infectious MLV particles. The infectivity of these particles, similar to what we reported before, was significantly reduced in a PSGL-1 dose-dependent fashion (Fig. 1d). The effect of this inhibition is comparable to that of SERINC5 (Fig. S1b). Correspondingly, we detected that PSGL-1 is released into the media from the transfected cells, likely within the released MLV particles (Fig. 1e). To further confirm, we performed Opti-prep ultracentrifugation to purify the MLV particles and detected that both mouse and human PSGL-1 co-migrated with MLV particles (Fig. 1f). These data, in combination with our previously reported results, including electro-microscopy to show the specific localization of PSGL-1 in the budding virions (12), strongly support that mouse PSGL-1 is packaged in the viral particles given the highly conserved sequence and function of PSGL-1 in mouse and human. In sum, the antiviral activities of PSGL-1 are conserved in mice for restricting MLV.

### PSGL-1 is degraded in a glycogag/glycoMA-dependent fashion

**Fig 2:**
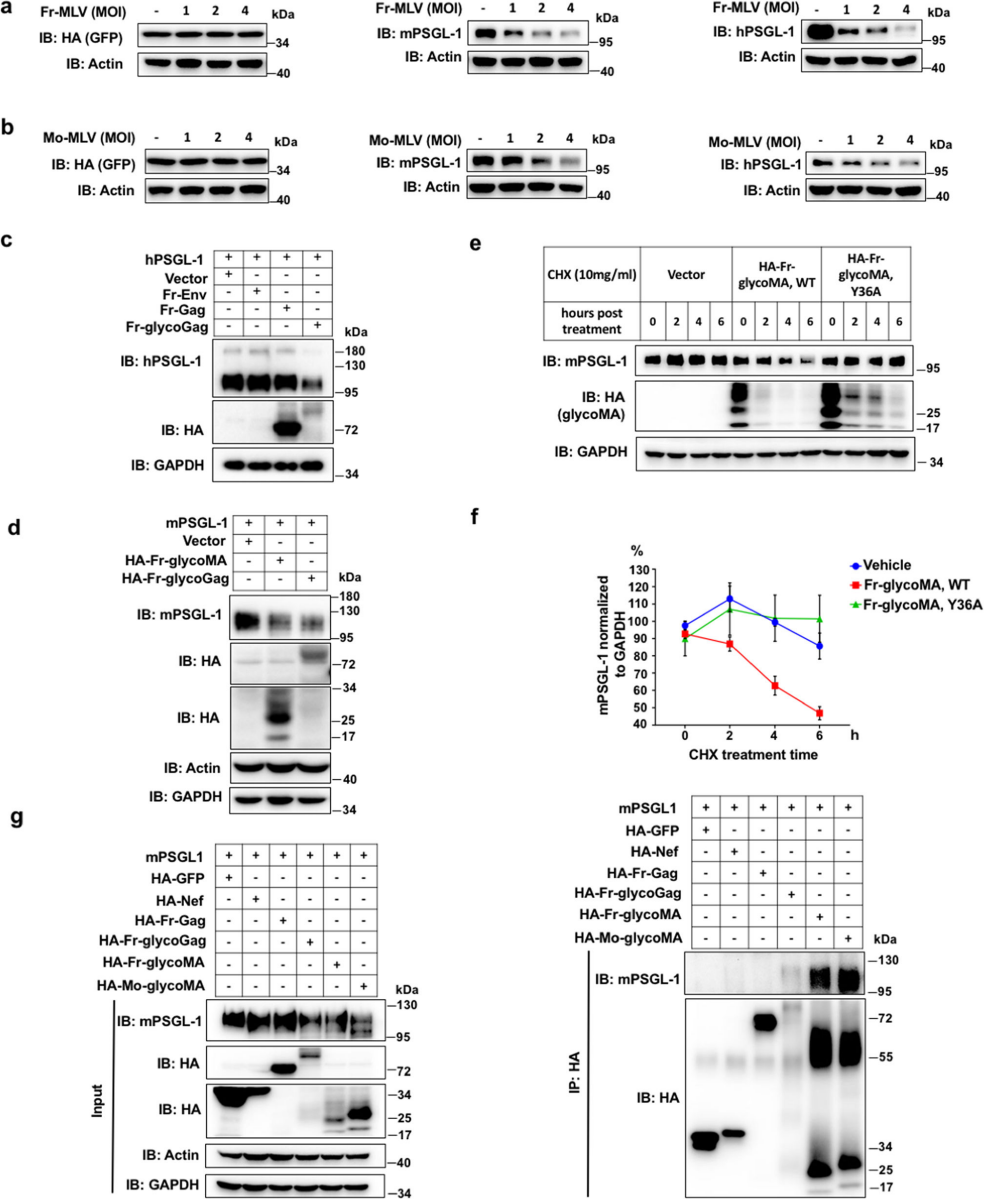
MLV glycoGag/glycoMA can induce PSGL-1 degradation. (a) Western blots of lysates of 3T3 cells overexpressing GFP (left), mPSGL-1 (middle), or hPSGL-1 (right) and infected by an increasing multiplicity of infection (MOI) of Fr-MLV. (b) Western blots of lysates of 3T3 cells overexpressing GFP (left), mPSGL-1 (middle), or hPSGL-1 (right) and infected by an increasing MOI of Mo-MLV. (c) Western blots of lysates of 293T cells co-transfected with plasmids expressing mPSGL-1 and one of the following hemagglutinin (HA)-tagged Fr-MLV proteins: Env, Gag, glycoGag, or an empty vector. Among these Fr-MLV proteins, the band of Env did not show likely because of that the heavy glycosylation of Env blocked the recognition of the HA tag by the antibody. Env overexpression was confirmed using RT-qPCR as shown in Fig. S2b. (d) Western blots of lysates of 293T cells co-transfected with plasmids expressing mPSGL-1 and one of the following Fr-MLV proteins: glyoGag, glycoMA, or an empty vector. The bands of glycoMA can be detected in the range from 17 to 34 kDa because of different levels of glycosylation, the main band of glycoMA is about 25 kDa. (e) 293T cells were co-transfected with plasmids expressing mPSGL-1 and glycoMA, glycoMA Y36A mutant, or an empty vector. One day after transfection, cells were treated with CHX and collected at the indicated time points. (f), Quantitative analysis of PSGL-1 levels in experiments shown in (e). (g) 293T cells were co-transfected with plasmids expressing mPSGL-1 and one of the following HA-tagged proteins: GFP, HIV-Nef, Fr-Gag, Fr-glycoGag, Fr-glycoMA, or Mo-glycoMA. Two days after transfection, the cells were harvested and incubated with beads conjugated with anti-HA antibody, then the input or IP products were analyzed by Western blots. The results of (a–d), (g) are representative of at least three independent experiments. The results of (e) are derived from two independent experiments.

Since PSGL-1 is antagonized by HIV-1 Vpu through the ubiquitin-proteasome degradation pathway (11), we wondered whether PSGL-1 is also degraded by MLV. Indeed, we found that Fr-MLV infection of 3T3 cells stably expressing exogenous mouse or human PSGL-1 caused the degradation of PSGL-1 in a viral-titer-dependent manner (Fig. 2a). As two controls, stably expressed GFP protein or endogenous housekeeping protein beta-actin is not affected by the infection (Fig. 2a). Mo-MLV infection had similar effects on human and mouse PSGL-1 protein levels (Fig. 2b). Importantly, the mRNA level of PSGL-1 was not changed due to the infection, suggesting the reduced protein level is due to post-transcriptional regulation (Fig. S2a). Next, we tested three Fr-MLV proteins individually, namely Env, Gag, and glycoGag. Only glycoGag expression led to a significant decrease in mPSGL-1 (Fig. 2c; Fig. S2b). In addition, the N-terminal portion of glycoGag, namely glycoMA, is sufficient to induce the decrease in the protein level of mPSGL-1 (Fig. 2d). We used a classic protein stability test, cycloheximide chase experiment, to show that this decrease in mPSGL-1 level is due to compromised protein stability, confirming that glycoMA promotes mPSGL-1 degradation (Fig. 2e and f; Fig. S2c). In this experiment, we used two controls, one is an empty vector, and another is a mutant glycoMA, Y36A, which lost the Nef-like effect and the activity of degradation of SERINC5 (Fig. S3a) (17, 18). This mutation also lost the ability to degrade PSGL-1, suggesting a similar degradation mechanism as that of SERINC5. Furthermore, this degradation is associated with a binding between glycoGag/glycoMA with mPSGL-1 as shown by a co-immunoprecipitation experiment. As controls, GFP, Fr-MLV Gag, or HIV Nef do not bind mPSGL-1, but the glycoMA of Fr-MLV and Mo-MLV can efficiently bind to mPSGL-1 and decrease its protein level. The co-immunoprecipitation (IP) of mPSGL-1 by glycoGag is observable but weaker than glycoMA, likely due to a weaker expression of glycoGag or a weaker binding between the HA-tagged Fr-glycoGag and HA-beads (Fig. 2g).

### GlycoMA induces K63 ubiquitination and lysosomal degradation of PSGL-1

**Fig 3:**
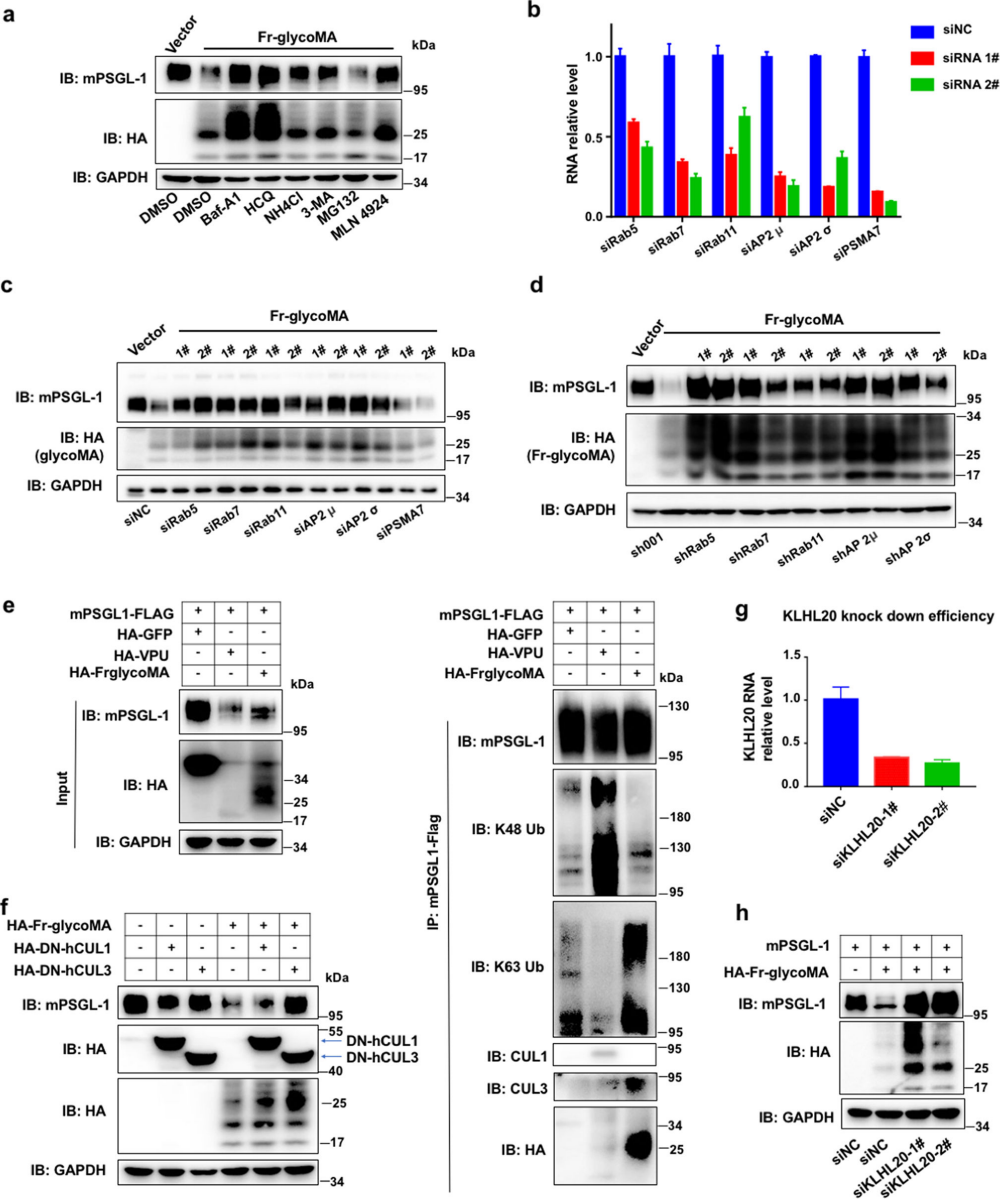
MLV glycoGag/glycoMA degrades PSGL-1 through the autophagy/lysosomal pathway. (a) HEK293T cells were co-transfected with mPSGL-1 or Fr-glycoMA or an empty vector for 48 h, hydroxychloroquine (HCQ), NH4Cl, 3-MA, and MLN 4924 were added 24 h post-transfection, and bafilomycin (BFA), MG132 was added 8 h before the cells were harvested. Then the cell lysates were analyzed by Western blotting. (b and c) HEK293T cells were transfected with siRNAs targeting indicated genes or non-targeting control siRNA, after 24 h the cells were co-transfected with mPSGL-1 and glycoMA for 48 h, then analyzed by RT-qPCR and Western blotting. An empty vector was used as a negative control for the glycoMA-expressing vector. (d) HEK293T cells stably expressing indicated shRNAs were co-transfected with mPSGL-1 and glycoMA for 48 h and analyzed by Western blotting. An empty vector was used as a negative control for the glycoMA-expressing vector. (e) HEK293T cells were co-transfected with a plasmid-expressing FLAG-tagged mPSGL-1 and one of the following HA-tagged proteins: GFP, HIV-Vpu, and Fr-glycoMA. Two days after transfection, the cells were lysed and incubated with anti-FLAG beads, then the input or IP products were analyzed by Western blots with indicated antibodies. (f) Western blots of lysates of HEK293T cells transfected with plasmids expressing mPSGL-1 and DN-CUL1 or DN-CUL3 or an empty vector. The Fr-glyoMA expressing plasmid (+) or an empty vector (−) is also transfected as indicated. (g and h) HEK293T cells were transfected with three siRNAs targeting KLHL20 or a non-targeting siRNA (NC). One day after transfection, cells were then transfected with plasmids expressing mPSGL-1 and Fr-glycoMA or an empty vector. Two days after plasmid transfection, cells were harvested and analyzed by qRT-PCR (f) and Western blotting (g). The results of (a–h) are representative of at least three independent experiments.

We further tested the mechanism of GlycoMA-induced mPSGL-1 degradation. Inhibitors of lysosomal or proteasomal degradations as well as the CRL E3 ligase family inhibitor MLN4924 were applied to the cells overexpressing both mPSGL-1 and Fr-glycoMA. Apparently, four autophagosome-lysosome pathway inhibitors (Bafilomycin A, hydroxychloroquine, NH_4_Cl, and 3-MA) blocked glycoMA-induced mPSGL-1 degradation to different degrees (Fig. 3a), so did the CRL inhibitor MLN4924, but not the proteasome inhibitor MG132 (Fig. 3a). This result is different from that of Vpu-induced hPSGL-1 degradation, which is mediated by the proteasome (11). We further confirmed with an RNAi strategy that the autophagosome-lysosome pathway is required for the glycoMA-induced mPSGL-1 degradation. We knocked down several key components in this pathway using siRNAs or shRNAs targeting Rab5, Rab7, Rab11, and AP2 (Fig. 3b; Fig. S4a). This knockdown rescued mPSGL-1 degradation by glycoGag or glycoMA to different degrees, as a control, siRNAs targeting a key proteasome component (PSMA7) did not rescue mPSGL-1 degradation, consistent with the rescue using small molecular inhibitors of these two degradation pathways (Fig. 3c and d; Fig. S4b).

Since we have shown previously that human PSGL-1 degradation is mediated by Vpu-dependent ubiquitination in a K48 chain-specific fashion (11), we further performed immunoblotting of ubiquitin to test whether glycoMA also induces mPSGL-1 ubiquitination. This experiment indeed showed that mPSGL-1 is ubiquitinated, however, in a K63 chain-specific manner (Fig. 3e). As a control, Vpu can promote K48 chain type but not K63 chain type ubiquitination of mouse PSGL-1 (Fig. 3e). The different ubiquitin chain types between Vpu- or glycoMA- induced ubiquitination likely determined the different degradation pathways, with K48 type leading to proteasomal degradation, while K63 type leading to lysosomal degradation. These results are consistent with many studies of cellular protein substrates of the ubiquitination pathway (19). In addition, these results further confirmed the interaction of mPSGL-1 with glycoMA, using a reciprocal pulldown strategy complementary to that shown in Fig. 2g (Fig. 3e). Recently, another restriction factor SERINC5 has been shown to be a ubiquitination substrate of Cul3-KLHL20 E3 ligase complex in a glycoMA-dependent fashion (20). We also tested whether this is the case for PSGL-1. Indeed, first of all, glycoMA-induced mPSGL-1 degradation is blocked by overexpressing a dominant negative Cul3 N-terminal domain (DN-Cul3), but not by a dominant negative Cul1 N-terminal domain (DN-Cul1) (Fig. 3f). This is opposite to what we observed for Vpu-induced hPSGL-1 degradation, which is blocked by DN-Cul1, but not DN-Cul3 (11). Furthermore, siRNA-mediated knockdown of KLHL20 also blocked glycoMA-mediated mPSGL-1 degradation (Fig. 3g and h). Interestingly, glycoMA was also stabilized by siRNA-mediated knockdown of KLHL20, consistent with the auto-ubiquitination of glycoMA by associating with Cul3-KLHL20 E3 ligase, a common phenomenon of E3 substrate receptor (Fig. 3h). Together, these data support that glycoMA recruits the Cul3-KLHL20 E3 ligase to catalyze the formation of K63 ubiquitin chain linked to mPSGL-1, which leads to the subsequent degradation of mPSGL-1 through the autophagosome-lysosome pathway.

### PSGL-1 underwent positive selection associated with Vpu/glycoMA binding and induced degradation

**Fig 4:**
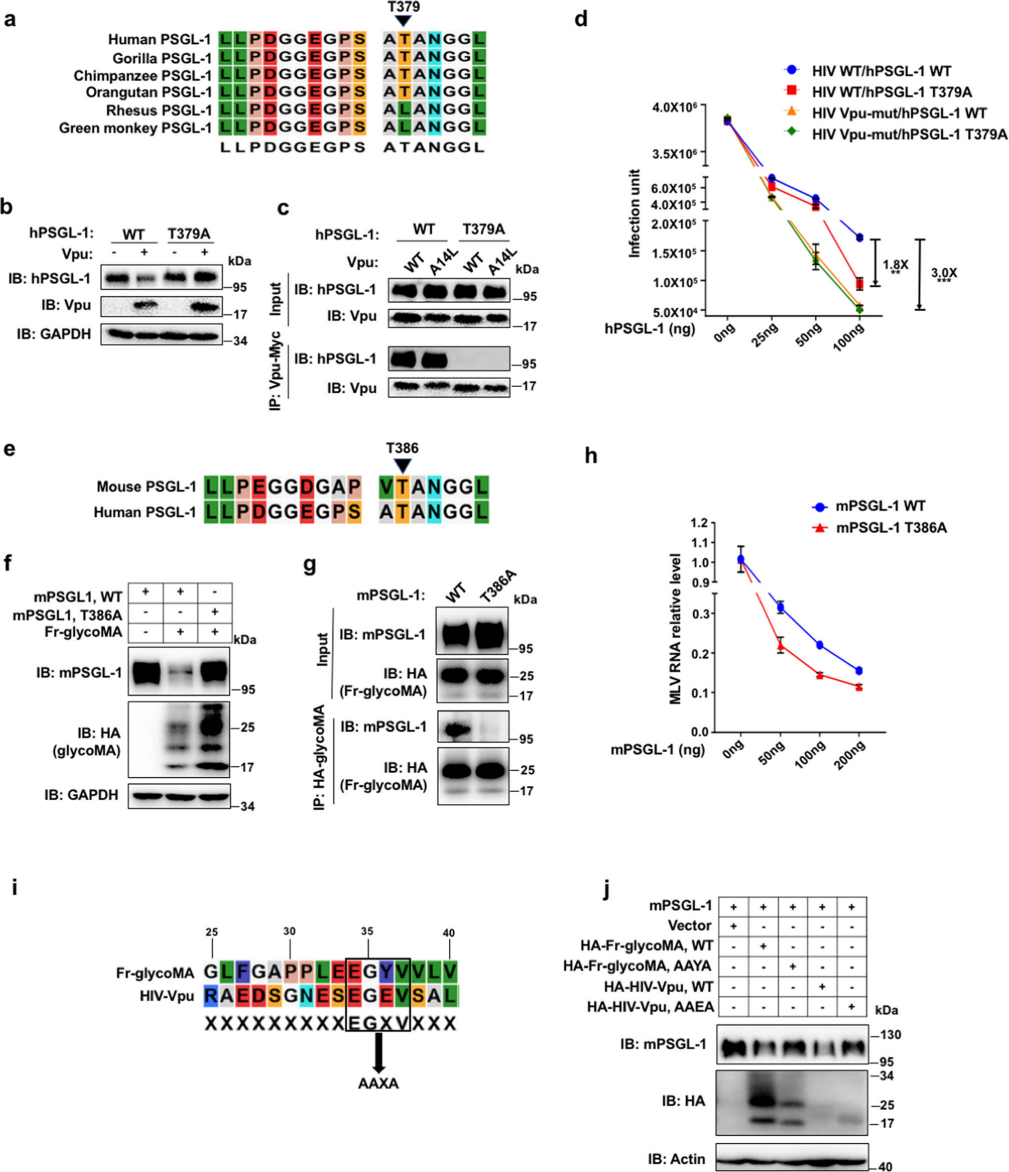
PSGL-1 underwent positive selection at residues associated with Vpu- and glycoMA binding. (a) Alignment of part of PSGL-1 sequences of six primate species showing positive selected residue of T379 in humans. (b) Western blots of lysates of 293T cells that were co-transfected with wild type (WT) or T379A hPSGL-1 and HIV-Vpu or an empty vector. (c) HEK293T cells were transfected with plasmids expressing WT or T379A hPSGL-1. A different batch of HEK293T cells were transfected with plasmids expressing Myc-Vpu or Myc-Vpu A14L. Two days after the transfection, the two batches of cell lysates were mixed as indicated and Vpu was immunoprecipitated with anti-Myc beads. The cell lysates and the precipitated proteins were analyzed by Western blotting. Myc-Vpu A14L is a mutant of Vpu that abolishes the degradation of tetherin induced by the Vpu. (d) HEK293T cells in six-well plates were co-transfected with 1 µg per well of pNL4-3 (HIV WT) or pNL4-3 Vpu-mut (HIV Vpu-mut) plasmid and plasmids expressing hPSGL-1 WT or hPSGL-1 T379A at the indicated dosages. Two days after transfection, the supernatants were collected and normalized for the p24 amount to infect TZM-bl cells for 48 h, and the infection units were measured with the β-galactosidase assay. (e) Alignment of part of PSGL-1 sequences of mouse and human showing T386 of mPSGL-1 corresponding to T379 of hPSGL-1. (f) Western blots of lysates of HEK293T cells co-transfected with WT or T386A mPSGL-1 and Fr-glycoMA or empty vector. (g) HEK293T cells were transfected with plasmids expressing WT or T386A mPSGL-1. A different batch of HEK293T cells was transfected with plasmids expressing HA-tagged Fr-glycoMA. Two days after the transfection, the two batches of cell lysates were mixed as indicated and glycoMA was immunoprecipitated anti-HA beads. The cell lysates and the precipitated proteins were analyzed by Western blotting. (h) HEK293T cells in six-well plates were co-transfected with 1 µg per well of pNCS (Mo-MLV) plasmid and plasmids expressing luciferase (0 ng), WT or T386A mPSGL-1 at the indicated dosages. Two days after transfection, the supernatants were collected and normalized for the MLV RNA copies amount to infect 3T3 cells for 48 h, and then RNA levels of Mo-MLV were detected by RT-qPCR. (i) Alignment of part of Fr-glycoMA and HIV-1 Vpu showing a conserved EGXV motif, the numbering corresponds to the amino acids of Fr-glycoMA. (j) Western blots of lysates of HEK293T cells co-transfected with mPSGL-1 and WT Fr-glycMA, AAXA mutant Fr-glycoMA or empty vector. The results of (a–j) are representative of at least three independent experiments.

Given that genes encoding restriction factors often undergo positive selections during evolution due to the high selection pressure exerted by the viruses, we examined the nucleotide sequences of PSGL-1 by multiple sequence alignment across six mammalian species (human, chimp, macaque, dog, mouse, and rat) extracted from a previous study (2, 21). We estimated the omega (d_N_/d_S_) values across all codons extracted from the M8 model from the PAML package (22). Over 40 sites demonstrated a d_N_/d_S_ value higher than 1, indicating positive selection (Fig. S5). The short intracellular domain (~60 amino acids) contains five sites with d_N_/d_S_ > 1, among which one site (threonine 379 of human PSGL-1 or threonine 386 of mouse PSGL-1) is outstanding with d_N_/d_S_ > 3 (Fig. 4a; Fig. S5). We found that the intracellular domain of hPSGL-1 is sufficient for binding with Vpu and critically a mutation of the positively selected T379 residue into alanine abolished this binding as well as Vpu-induced PSGL-1 degradation of hPSGL-1 (Fig. 4b and c). This binding of Vpu to hPSGL-1 is distinct from that of Vpu to tetherin, as the A14L mutation of Vpu, known to abolish tetherin binding, did not affect hPSGL-1 binding (Fig. 4c). This T379A mutation of hPSGL-1 further enhanced its antiviral activity to restrict progeny virions, presumably due to the resistance to Vpu-mediated degradation (Fig. 4d). Similarly, mutation of the T386 residue of mouse PSGL-1, which is homologous to the T379 residue of human PSGL-1 (Fig. 4e), also abolished the binding with glycoMA and the induced degradation of mPSGL-1 (Fig. 4f and g). Consistently, this mutation also enhanced the restriction activity of mPSGL-1 (Fig. 4h). We also observed that Vpu can degrade mPSGL-1, which is abolished by the T386A mutation of mPSGL-1 (Fig. S6a). Similarly, glycoMA can also degrade hPSGL-1, which is also abolished by the T379A mutation of hPSGL-1 (Fig. S6b). These data suggest that this positively selected threonine of PSGL-1 is a key residue mediating binding with Vpu or glycoMA. These data also suggest that Vpu and glycoMA share a similar mechanism of binding to PSGL-1. We compared the sequences of Vpu and glycoMA and identified a shared EGXV motif (X indicates any amino acid) (Fig. 4i). Mutation of this motif in mPSGL-1 to AAXA abolished the glycoMA- or Vpu-induced degradation of mPSGL-1, supporting the importance of the EGXV motif in binding with PSGL-1 (Fig. 4j).

### PSGL-1 knockout renders mice more susceptible to MLV infection

**Fig 5:**
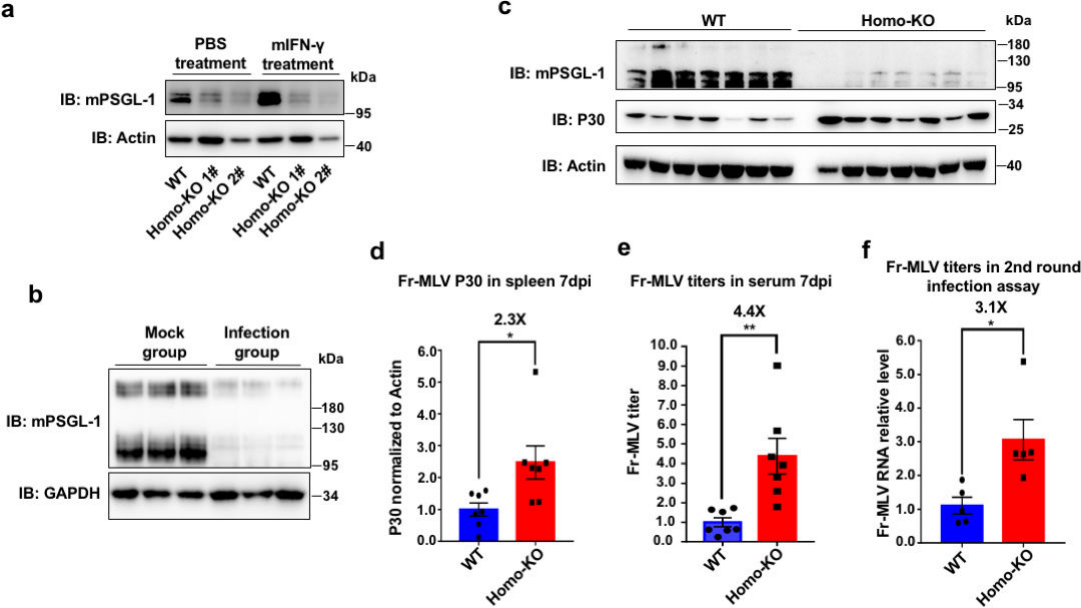
PSGL-1 knockout in mice promotes the infectivity of Fr-MLV. (a) CD4 +T cells isolated from WT or PSGL-1 knockout littermates were treated with IFN-γ or PBS for 48 h, then the cell lysates were analyzed with Western blot. (b) BALB/c mice infected with Fr-MLV through intraperitoneal injection with 300 multiplicity of infection (MOI) were sacrificed at 14 days post-infection (dpi). The spleens were harvested and homogenized and lysed with radioimmunoprecipitation assay buffer and analyzed by Western blot. (c and d) WT and PSGL-1 knockout littermates were infected with 50 MOI of Fr-MLV, 7 dpi the mice were sacrificed and the spleens were harvested and analyzed with Western blot. Results of the Western blot were analyzed with Image-J, and the intensity of P30 was normalized to that of actin. (e) The RNA levels of Fr-MLV isolated from the sera of mice as in (d) were measured by RT-qPCR. (f) The RNA levels of Fr-MLV isolated from the spleen of infected WT or PSGL-1 homo-KO mice were normalized by RT-qPCR, then viruses with equal amounts of RNA were used to infect naive 3T3 cells for 48 h, then the RNA levels of Fr-MLV in 3T3 cell were detected by RT-qPCR. The results of (a–f) are representative of at least three independent experiments.

Given evidence of the conservation of mouse PSGL-1’s antiviral activity, we sought to evaluate this activity *in vivo*. We generate PSGL-1 knockout mice using the CRISPR-Cas9 technology and confirmed the knockout using PCR and Western blot of blood samples (Fig. S7a and b; Fig. 5a). We also treated the mice and wild-type (WT) control mice with mouse IFN-γ and found that mPSGL-1 expression is induced by IFN-γ in the T cells of wild-type mice but not the knockout littermates (Fig. 5a), which is similar to that we observed in human CD4+T cells (11). We established the infection model of Fr-MLV in BALB/c mice, which is manifested in the enlarged spleens after infection (Fig. S8a). Importantly, we demonstrated a dramatic PSGL-1 degradation in the spleens post-infection (Fig. 5b). We then infected the two groups of mice and found that compared to wild-type littermates, the PSGL-1 knockout mice showed increased infection as measured by the Western blot of the p30 antigen levels of the spleens from the sacrificed mice (Fig. 5c and d) and the viral RNA levels in the serum (Fig. 5e). The enlargement of the spleens as measured by the spleen index (spleen weight/body weight) is moderately increased in the knockout mice, although not reaching a statistically significantly level (Fig. S8b). Nevertheless, we isolated MLV particles from the infected spleens, normalized the MLV from different mice by qRT-PCR and infected 3T3 cells. Consistent with *in vitro* experiments, MLV from PSGL-1 knockout mice spleens demonstrated significantly higher infectivity than those from wild-type mice, even after normalization of viral particles by RT-qPCR (Fig. 5f). Since PSGL-1 has been associated with T-cell survival after stimulation and PSGL-1 deficiency found to lead to increased T-cell survival (23), we checked the percentage of CD4+, CD8+, CD11a+, and CD49+ T cell populations in both uninfected and infected spleens, but found no obvious changes between WT and PSGL-1 null mice (Fig. S9a and b). Therefore, the reported effects on T-cell survival are unlikely to account for the increased MLV infectivity in PSGL-1 knockout mice.

**Fig 6:**
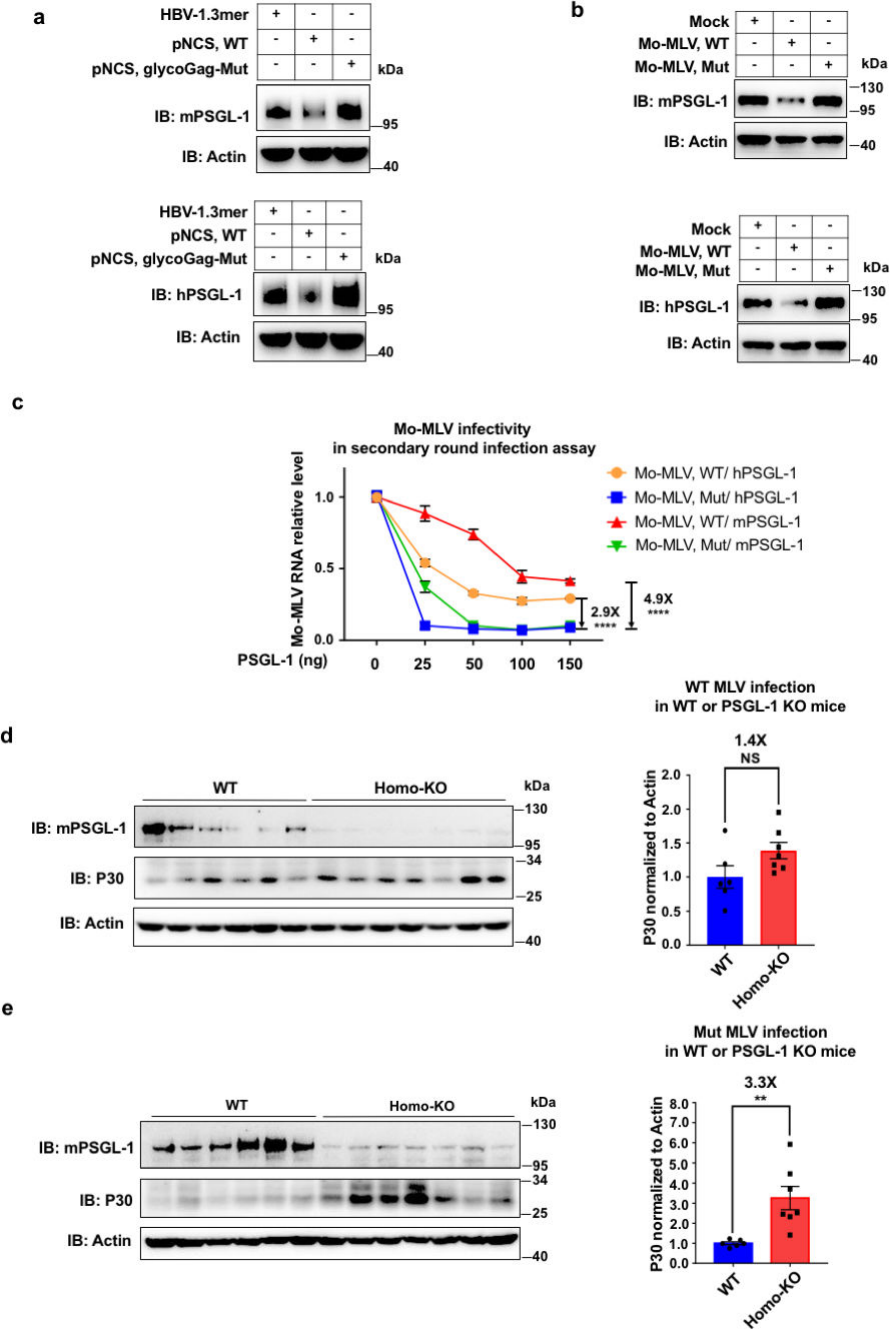
Mutation in glycoGag abolished the function of Mo-MLV to degrade PSGL-1 and affected the infectivity *in vitro* and *in vivo*. (a) Western blots of lysates of HEK293T cells co-transfected with mouse or human PSGL-1 plasmids (400 ng per well) and WT Mo-MLV producer plasmid (pNCS, WT) or Y36A/L39A mutants in glycoGag of Mo-MLV (pNCS, glycoGag-Mut) and HBV-1.3mer plasmids (200 ng per well) as a control. (b) Western blots of lysates of 3T3 stable cell lines that express mouse or human PSGL-1 infected with 5 multiplicity of infection (MOI) of Mo-MLV, WT (produced from pNCS, WT) or Mo-MLV, Mut (produced from pNCS, glycoGag-Mut). (c) 3T3-naive cells were infected with the 1 MOI progeny virus produced from co-transfection with the Mo-MLV producer plasmid pNCS, WT or pNCS, glycoGag-Mut and mouse or human PSGL-1 expressing plasmids in HEK293T cells for 48 h, then the cells were harvested and Mo-MLV RNA levels were quantitated by RT-qPCR. (d) WT and PSGL-1 knockout littermates were infected with 5 MOI of Mo-MLV, WT within 24–36 h after birth. At 14 dpi, the mice were sacrificed and the spleens were harvested and analyzed with Western blot (left panel). Results of the Western blot were analyzed with Image-J, and the intensity of P30 was normalized to that of actin (right panel). (e) WT and PSGL-1 knockout littermates were infected with 5 MOI of Mo-MLV, Mut within 24–36 h after birth. At 14 dpi, the mice were sacrificed and the spleen was harvested and analyzed with Western blot (left panel). Results of the Western blot were analyzed with Image-J, and the intensity of P30 was normalized to that of actin (right panel). The results of (a–e) are representative of at least three independent experiments.

To further demonstrate the antagonism between glycoGag and PSGL-1, we used Mo-MLV, for which a molecular clone is available. In previous studies, it was shown that P31A, Y36A, L39A, and R63A mutants within the conserved Y_36_XXL_39_ motif of glycoGag abolished the degradation of SERINC5 (Fig. S3a) (17, 18). We confirmed those residues are also crucial for PSGL-1 downregulation (Fig. 2e; Fig. S3b and c). We generated the Y36A/L39A mutant virus and showed that this mutant can no longer degrade mPSGL-1 or hPSGL-1, which is true for plasmids transfection experiments and virus infection (Fig. 6a and b). We also found that the viruses packaged from transfection of 293T cells demonstrated reduced infectivity in a PSGL-1 dose-dependent fashion (Fig. 6c). Importantly, the mutant virus displayed a much steeper decline curve of infectivity than the wild-type virus, consistent with a loss of the ability of the mutant virus to antagonize PSGL-1. We used the viruses to infect mice littermates of wild-type PSGL-1 or PSGL-1 knockout genotypes. We found that while the wild-type virus showed a slightly higher infectivity in PSGL-1 knockout mice than in the wild-type littermates (Fig. 6d), in contrast, the Y36A/L39A mutant virus demonstrated a much higher infectivity in the PSGL-1 knockout than in the wild-type mice (Fig. 6e). These results confirm that PSGL-1’s antiviral activity is mitigated by glycoGag; therefore, the loss-of-function mutation of glycoGag would manifest the restriction activity of PSGL-1. These data from mouse experiments demonstrated the antiviral role of PSGL-1 *in vivo*, further proving that PSGL-1 is an evolutionarily conserved restriction factor.

## DISCUSSION

The studies of HIV restriction factors have summarized four common properties shared by most of these antiviral genes: potent antiviral capacity, inducible by interferons, counteracted by HIV, and positively selected during evolution (8). Here we demonstrate that PSGL-1 fully meets the four properties and these qualifications are conserved from mouse to human. Similar to human PSGL-1, the mouse version demonstrates both early and late inhibition of MLV in murine 3T3 cells. Importantly, the knockout of PSGL-1 in mice promotes the infection of MLV *in vivo*. IFN-γ also induces mouse PSGL-1 expression, consistent with that of human PSGL-1. We further show here that mouse PSGL-1 is counteracted by MLV glycoGag/glycoMA via ubiquitination and subsequent degradation. This degradation is also conspicuous in a mouse infection model. Mechanistically distinct from Vpu, which recruits Cul1-Rbx1-Skp1^βTrCP2^ E3 ligase (SCF^βTrCP2^), glycoGag/glycoMA recruits Cul3-based E3 ligase Cul3-Rbx1-KLHL20 (CRL3^KLHL20^). Consequently, glycoGag/glycoMA-mediated ubiquitination is different from that mediated by Vpu in that the K63 type chain is formed by the CRL3^KLHL20^-glycoGag/glycoMA complex, while the K48 type ubiquitin chain is formed by the SCF^βTrCP2^-Vpu complex. This difference in ubiquitin chain type likely results in the different pathways of PSGL-1 degradation, with the K63 chain leading to lysosomal degradation while the K48 chain to proteasomal degradation. GlycoGag/glycoMA employs a similar strategy to counteract SERINC5 and PSGL-1 by hijacking the CRL3^KLHL20^ ligase. It is not surprising for an E3 ligase to target multiple substrate proteins for ubiquitination and degradation, as we have extensively reviewed in a recent review article (10). While for APOBEC3, glycoGag might utilize a totally different mechanism to counteract, showing remarkable functional versatility (24). Importantly, the counteracting measure against PSGL-1 is conserved between MLV and HIV-1, supporting that PSGL-1 is a highly conserved restriction factor in mammals. The conservation of the antiviral function of PSGL-1 is associated with the positive selection of certain key amino acids of PSGL-1, which mediates its binding with the Vpu/glycoMA. We identified T379 of hPSGL-1 or T386 of mPSGL-1 as such a residue that mediates Vpu/glycoMA binding. Mutation of this residue can lead to the abolishment of Vpu/glycoMA binding and subsequent degradation. The fact that this amino acid is one of the three most highly selected sites supports the evolutionary pressure from the antagonism between PSGL-1 and Vpu/glycoMA. Meeting the four qualifications of HIV restriction factors, PSGL-1 should therefore be considered a *bona fide* restriction factor with broad-spectrum antiviral activities. PSGL-1 has multiple functions in the immune system, including tethering leukocytes to endothelial of the inflammation sites (25) and functioning as a receptor for enterovirus 71 (26). More recently, PSGL-1 has been shown to function as an immune checkpoint molecule that can promote T-cell exhaustion (23). Considering these different roles besides being a restriction factor, there could be multiple benefits for HIV or MLV that infect the lymphocytes to degrade PSGL-1 proteins. For example, this degradation might promote the trafficking of infected lymphocytes beyond inflammatory sites given the well-established role of PSGL-1 in the tethering of T cells at these sites (25), which might help the transmission of the viruses with the lymphocytes. PSGL-1 degradation might also promote T-cell activation (23), thereby generating more substrates for HIV/MLV infection. In the same vein of reasoning, a drug developed to block PSGL-1 degradation might achieve multiple therapeutic roles to treat the infection and associated pathology.

## MATERIALS AND METHODS

### Mouse husbandry and infections

All mice were housed and bred according to the policies of the Institutional Animal Care and Use Committee of the Tsinghua University. The experiments performed with mice in this study were approved by this committee. BALB/c mice were obtained from Beijing Vital River Laboratory Animal Technology Co., Ltd. For *in vivo* Mo-MLV infection experiments, mice were infected by intraperitoneal (i.p.) injections with 5 multiplicity of infection (MOI) of Mo-MLV virus stock within 24–36 h after birth. Spleens from infected mice were harvested 14 days post-infection and used for the detection of P30 level by Western blot. For *in vivo* Fr-MLV infection experiments, 6- to 8-week-old mice were infected by i.p. injections with 50 MOI of Fr-MLV stock. Spleens from infected mice were harvested 7 days post-infection and used for the detection of P30 level by Western blot.

### Generation of PSGL-1^−/−^ mice

We used the CRISPR/Cas9 technology to generate the PSGL-1^-/-^ mice. The second exon of PSGL-1 was targeted by three guide RNAs (gRNAs) [gRNA1 (5′-GAGAGAGATTCCACGAGCGC-3′), gRNA2 (5′-TGGGGGAGTAGTTCCGCACT-3′), and gRNA3 (5′-TCTGTGTTATACGTATAGTC-3′)]. The gRNAs and Cas9 mRNA were transferred into zygotes of BALB/c mice by microinjection. DNA from the mice generated from the micro injection was sent for sequencing. One founder line was discovered to have a sizable 767 bp deletion that included part of exon 2 (158–924 bp). For the purpose of genotyping, we used three primers shown below. The first and second primers amplify the WT allele (the product is 554 bp), and the first and third primers amplify the deleted allele (the product is 245 bp).

Common forward primer: 5′-TTGGGGATGACGATTTTGAGGA-3′

WT reverse primer: 5′-GGGAAGCTGGGTGGTCTCTG-3′

KO reverse primer: 5′-AAGCTGTGCAGGGTGAGGTC-3′

The PSGL-1^-/-^ mice developed normally and show no significant difference with wild-type littermates, as measured by weight, behavior, and fetility.

### Cells and cell culture

HEK293T cells and NIH/3T3 cells (from ATCC) were cultured in Dulbecco’s Modified Eagle Medium (DMEM) supplemented with 10% heat-inactivated fetal bovine serum (FBS), 6 mM L-glutamine, penicillin (100 U/mL), and streptavidin (100 μg/mL). Splenocytes were isolated from WT and PSGL-1 homo-KO mice, and CD4 T cells were purified from splenocytes using anti-Mouse CD4+PerCP/Cyanine5.5-tagged antibody (BioLegend, 100539) by fluorescence-activated cell sorting (FACS), and then cultured in RPMI supplemented with 10% heat-inactivated FBS), 6 mM L-glutamine, penicillin (100 U/mL), and streptavidin (100 μg/mL). Naive CD4 T cells were activated with CD63 and CD81 antibody (Abcam 193349, 109201) and recombinant murine IL-2 (8 μg/mL, Peprotech, 212–12) for 72 h.

### Plasmids

For exogenous expression in cell lines, mammalian cells, genes encoding mouse, or human PSGL-1, GFP and luciferase were cloned in a pLenti-CMV vector with an N-terminal HA tag and FLAG tag. The MLV genes of Gag, glycoGag, glycoMA, and Env were cloned from pNCS or synthesized following the sequence of Z11128.1 (GeneBank) and inserted into the pLenti-CMV vector. The HIV genes of Nef and Vpu were cloned from pNL4-3 and inserted into the pLenti-CMV vector. For immunoprecipitation assay, mouse or human PSGL-1 was cloned into a pcDNA3.1 vector with a C-terminal FLAG tag; Gag, glycoGag, glycoMA of MLV, Nef, Vpu of HIV, and GFP were cloned into a pCAG vector with N-terminal HA tag. Point mutations were introduced by site-directed mutagenesis in the backbone of pcDNA3.1, and followed by digestion of parental DNA with DpnI.

### Plasmid transfection assay

HEK293T cells were seeded in the amount of 0.2 million cells per well of a 12-well plate. After 16 h, the plasmids were transfected into HEK293T cells using Chemifect transfection reagent following the manufacturer’s protocol (Beijing Fengrui Biology Technology). For protein interaction assay, PSGL-1 plasmids were transfected with 400 ng per well, and plasmids of viral proteins or controls were transfected with 200 ng per well. At 48 h post-transfection, the cells were harvested for Western analysis or RT-qPCR.

### Antibodies and beads

The following antibodies were used for Western Blotting: anti-mPSGL-1 (1:1,000; 557787, BD Pharmingen), anti-hPSGL-1 (1:750; sc-13535, Santa Cruz), anti-HA (1:1,000; 901514, Biolegend), anti-Myc (1:1,000; AT0023-2, CMCTAG), anti-P30 (1:1,000; ab130757, Abcam), anti-ubiquitin K48 (1:1,000; ab140601, Abcam), anti-ubiquitin K63 (1:1,000; 14–6077-82, Thermofisher), anti-Actin (1:10,000; BE0022, EASYBIO), and anti-GAPDH (1:5,000; BE0023, EASYBIO). For immunoprecipitation assay, anti-FLAG magnetic beads (HY-K0207, MCE), anti-HA magnetic beads (HY-K0201, MCE), anti-Myc magnetic beads (B26301, Bimake) were used.

### Western blot analysis

The cell samples or animal tissues were lysed with the radioimmunoprecipitation assay (RIPA, Beyotime, P0013C) lysis buffer. Then cell samples were normalized by cell number, or animal tissue samples were normalized by total protein measured by bicinchoninic acid (BCA) method (Beyotime, P0009), separated in a 10% SDS/PAGE gel. Western blotting was run with 0.01% SDS in transfer buffer for PSGL-1 protein transfer from the gel to polyvinylidene difluoride membrane and detected with corresponding antibodies listed above.

### Virus preparation and passage

The Mo-MLV producer plasmid of pNCS was a gift from Guangxia Gao (Chinese Academy of Sciences), strain of Fr-MLV was a gift from Xiaolan Cui (China Academy of Chinese Medical Sciences). Mo-MLV used to one-round infection assay *in vitro* or newborn mice infection *in vivo* were produced by transfection of 293T cells in a 10 cm dish with 10 µg plasmids of pNCS for 48 h, then the supernatants were harvested, filtered through 0.22 μm, and stored at −80°C. Mo-MLV used to secondary-round infection assay *in vitro* was produced by transfection of 293T cells in a six-well plate with 2 µg plasmid of pNCS and PSGL-1 expression plasmid at the indicated doses for 48 h, then harvested and filtered through 0.22 μm, and stored at −80°C. For Fr-MLV passage in BALB/c mice, BALB/c mice were infected with 300 MOI spleen suspension by intraperitoneal injection, 14 days post-infection the mice were sacrified and the spleens were collected. Then the spleen was homogenized with nine times of volume of DMEM, filtered through 0.22 μm, and stored at −80°C. The titers of Mo-MLV and Fr-MLV were determined by RT-qPCR.

### Cell infection assays

NIH/3T3 cell clones stably expressing mouse or human PSGL-1 were generated by retroviral transduction. For *in vitro* replication assays, NIH/3T3 stable expressing cells mentioned above were infected by adding various titers of viruses as indicated. To detect MLV RNA products, cells were harvested 48 h post-infection. To detect late reverse transcription products and 2-LTR circles, and cells were harvested 24 h post-infection.

### FACS analysis

Blood and spleens were collected from PSGL-1^+/+^ and PSGL-1^−/−^ mice of similar ages. After lysis of red blood cells, the PBMC and splenocytes were stained with the antibody of CD4 (RM4-5)/Percy-cy5.5 (BioLegend, 100539), CD8a (53–6.7)/FITC (BioLegend, 100705), CD11a (M17/4)/APC (BioLegend, 101119), and CD49d (R1-2)/PE (BioLegend, 103607). Stained cells were processed using BD LSRFortessa. Results were analyzed using FlowJo software.

### shRNA and siRNA

All of the shRNAs were bought from Sigma, and all of the siRNAs were purchased from JTSBIO. The shRNAs were cloned into the lentiviral vector pLKO.1. For siRNA transfection, Lipofectamine RNAiMax (Invitrogen) was used following the manufacturer’s protocol, and the knockdown efficiency was qualified by qRT–PCR 24 h after transfection.

The shRNAs sequences used in this study are as below:

shNC: CGTGATCTTCACCGACAAGAT

shRab5-1#: GCAGCCATAGTTGTATATGAT

shRab5-2#: CCAGGAATCAGTGTTGTAGTA

shRab7-1#: GCCACAATAGGAGCTGACTTT

shRab7-2#: CAACGAATTTCCTGAACCTAT

shRab11-1#: CCTGTCTCGATTTACTCGAAA

shRab11-2#: GAGCTATAACATCAGCATATT

shAP-2μ−1#: GACGCCAAACACACCAACTTT

shAP-2μ−2#: GCCAAGTGGTACATGCAGTTT

shAP-2σ−1#: GTGGTCATCAAGTCCAACTTT

shAP-2σ−2#: CACCAGCTTCTTCCACGTTAA

The siRNA sequences used in this study are as below:

siNC sense: UUCUCCGAACGUGUCACGUUU

siNC antisense: AAACGUGACACGUUCGGAGAA

siKLHL20-1# sense: GGUGGCGUAGGAGUUAUUAA

siKLHL20-1# antisense: UUAAUAACUCCUACGCCACC

siKLHL20-2# sense: GCCUGCUGUGAAUUCUUAA

siKLHL20-2# antisense: UUAAGAAUUCACAGCAGGC

siRab5a-1#: CCAACCAGGAATCAGTGTT

siRab5a-2#: AGGAATCAGTGTTGTAGTA

siRab7a-1#: GGGAGAUUCUGGAGUCGGGAAdTdT

siRab7a-2#: CCACAAUAGGAGCUGACUUdTdT

siRab11-1#: GCAACAAUGUGGUUUCCUAU

siRab11-2#: CAAGAGCGAUAUCGAGCUA

siAP2M-1#: AGUUUGAGCUUAUGAGGUAtt

siAP2M-2#: AGUUUGAGCUUAUGAGGUA

siAP2S-1#: CCGGAACUUUAAGAUCAUU

siAP2S-2#: GACCUGGUGUUCAACUUCU

siPSMA7-1#: GAGATCAATCCCTCAAGAT

siPSMA7-2#: AAGAAATTGAGAAGTATGT

### Immunoprecipitation assay

For the immunoprecipitation assay using anti-HA magnetic beads, 293T cells in each well of six-well-plates were co-transfected with 2 μg pcDNA3.1- mPSGL-1 and 0.5 μg-GFP, pcAG-Nef, pcAG-Fr-Gag, pcAG-Fr-glycoGag, pcAG-Fr-glycoMA, or pcAG-Mo-glycoMA individually. The cells were lysed with IP buffer (50 mM Tris-Cl, pH 7.5, 100 mM NaCl, 50 mM NaF, 1 mM EDTA, 0.5% NP-40, and protease inhibitor cocktail) 48 h after transfection. Then the cell lysates were incubated with 15 μL anti-HA magnetic beads overnight at 4°C. The beads were washed with IP buffer for five times the next day and boiled in 1× SDS-PAGE buffer at 95°C for 10 min. For the detection of PSGL-1 ubiquitination and E3 ligase components involved in the process of PSGL-1 due to glycoMA, cells in six-well plates were co-transfected 2 μg pcDNA3.1-FLAG-mPSGL-1 and 0.5 μg pcAG-GFP, pcAG-Vpu, pcAG-Fr-glycoMA. 48 h after transfection, cells were harvested and lysed with IP buffer, then the cell lysates were incubated with 15 μL anti-FLAG magnetic beads overnight at 4°C. The beads were washed with IP buffer for five times the next day and boiled in 1× SDS-PAGE buffer at 95°C for 10 min. For the detection of the binding between WT PSGL-1 or mutant PSGL-1 (hT379A, mT386A) and Vpu or glycoMA, one batch of 293T cells in six-well plates was transfected with 2 μg FLAG-tagged WT or mutant PSGL-1 plasmids, another batch of 293T cells in six-well plates was transfected with 0.5 μg Myc-Vpu or HA-glycoMA plasmids separately for 48 h. After cell harvest, the cells were lysed with IP buffer, two batches of cell lysates were mixed and incubated with 40 μL anti-Myc magnetic beads or 15 μL anti-Myc magnetic beads over night at 4°C. The beads were washed with IP buffer for five times the next day and boiled in 1× SDS-PAGE buffer at 95°C for 10 min.

### Real-time PCR amplification

Quantitative real-time PCR analyses of the following genes were carried out with the SYBR qPCR enzyme (Q321, Vazyme) and data were analyzed by the Bio-Rad CFX96 system. The primer sets were as follows:

Mouse GAPDH forward primer: 5′-AGGTCGGTGTGAACGGATTTG-3′

Mouse GAPDH reverse primer: 5′-TGTAGACCATGTAGTTGAGGTCA-3′

Human GAPDH forward primer: 5′-CGGAGTCAACGGATTTGGTCGTAT-3′

Human GAPDH reverse primer: 5'- AGCCTTCTCCATGGTGGTGAAGAC-3′

Mouse PSGL-1 forward primer: 5′-CCCTGGCAACAGCCTTCAG-3′

Mouse PSGL-1 reverse primer: 5′-GGGTCCTCAAAATCGTCATCC-3′

Mo-MLV forward primer: 5′-GCGCCAGTCCTCCGATTGACTG-3′

Mo-MLV reverse primer: 5′-CGGGTAGTCAATCACTCAG-3′

Fr-MLV forward primer: 5′-GGACAGAAACTACCGCCCTG-3′

Fr-MLV reverse primer: 5′-ACAACCTCAGACAACGAAGTAAGA-3′

Rab5 forward primer: 5′-AGACCCAACGGGCCAAATAC-3′

Rab5 reverse primer: 5′-GCCCCAATGGTACTCTCTTGAA-3′

Rab7 forward primer: 5′-GTGTTGCTGAAGGTTATCATCCT-3′

Rab7 reverse primer: 5′-GCTCCTATTGTGGCTTTGTACTG-3′

Rab11 forward primer: 5′-CAACAAGAAGCATCCAGGTTGA-3′

Rab11 reverse primer: 5′-GCACCTACAGCTCCACGATAAT-3′

AP-2μ forward primer: 5′-CTCATCTCCCGAGTCTACCGA-3′

AP-2μ reverse primer: 5′-GTTGGACCGCTTAACGTGGA-3′

AP-2σ forward primer: 5′-ATGATCCGCTTTATCCTCATCCA-3′

AP-2σ reverse primer: 5′-AAGTTGGTGTGTTTGGCGTCT-3′

KLHL20 forward primer: 5′-GTGATGGCCTGGGTCAAATAC-3′

KLHL20 forward primer: 5′-CTCTGCATTCTTCATCACTTT-3′

For the quantification of late reverse transcription products and 2-LTR circles, the following primers were used. The amplifications were carried out with the SYBR-Green qPCR enzyme (Q321, Vazyme) and data were analyzed by the Bio-Rad CFX96 system.

Mouse mitochondrial forward primer: 5′-TGCTAGCCGCAGGCATTAC-3′

Mouse mitochondrial reverse primer: 5′-GGGTGCCCAAAGAATCAGAAC-3′

MLV late RT forward primer: 5′-CGTCAGCGGGGGTCTTTC-3′

MLV late RT reverse primer: 5′-CTGGGCAGGGGTCTCCCG-3′

MLV 2LTR forward primer: 5′-CTCTTTTATTGAGCTCGGG-3′

MLV 2LTR reverse primer: 5′-AGTCCTCCGATTGACTGAG-3′

### Inhibitor treatments of cells

For all of the inhibitor experiments, the inhibitor dosages were first tested by Cell TiterGlo (Promega) to determine a non-toxic dose range. For treatment with the proteasomal and lysosomal inhibitors, HCQ/hydroxychloroquine sulfate (50 µM; S4430, Selleck), NH4Cl (20 µM; 12–0209, SINOPHARM), 3-MA/3-methyladenine (20 µM; S2767, Selleck), and MLN4924/Pevonedistat (20 µM; S7109, Selleck) were added 24 h post-plasmids transfection; Baf-A1/Brefeldin A (1 µM; S7046, Selleck) and MG132 (20 µM; S2619, Selleck) were added 8 h before cells were harvested. For the CHX chase experiment, CHX (10 µg/mL; C7698, Sigma) was added 24 h post-plasmid transfection. For the IFN-γ treatment experiments, cells were exposed to 10 μg/mL recombinant murine IFN-γ (315–05, Peprotech) for 48 h followed by cell lysis and Western blotting.

### Statistical analysis

All experiments have been repeated at least three times unless otherwise specified. All of the bar graphs are shown with the mean ± deviations. An unpaired, two-tailed *t*-test was used to calculate the *P* value unless otherwise specified. The *P* values are indicated as follows: * indicates *P* < 0.05, ** indicates *P* < 0.01, *** indicates <0.001, *** indicates *P* < 0.0001, and NS indicates not significant as *P* > 0.05.

## SUPPLEMENTAL MATERIAL

Supplemental figures mbio.00387-23-s0001.pdf Fig. S1-S9.

ASM does not own the copyrights to Supplemental Material that may be linked to, or accessed through, an article. The authors have granted ASM a non-exclusive, world-wide license to publish the Supplemental Material files. Please contact the corresponding author directly for reuse.
