# Supplementary material for: PSGL-1 is an evolutionarily conserved antiviral restriction factor
Source: mBio. 2023 Oct 3;14(5):e00387-23. doi: 10.1128/mbio.00387-23 (PMC10653843; doi:10.1128/mbio.00387-23)
Supplement: Supplemental figures — Fig. S1-S9. [file mbio.00387-23-s0001.pdf]

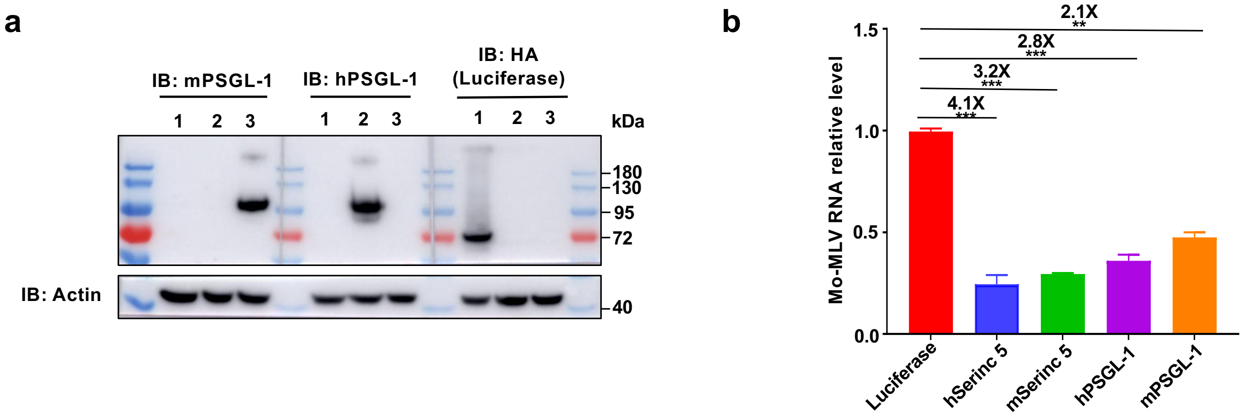

Supplementary Figure 1. a. Western blots of lysates of 3T3 stable cell lines expressing mPSGL-1 (Line 1), hPSGL-1 (Line 2) and HA tagged Luciferase (Line3). **b.** 293T cells in 6-well plates were co-transfected with 1 µg per well of pNCS (Mo-MLV WT) plasmid and 100ng plasmid expressing Luciferase, hSerinc 5, mSerinc 5, hPSGL-1 or mPSGL-1. Two days after transfection, the supernatants were collected and normalized for the MLV RNA copies amount to infect Rat2 cells for 48 h, and then RNA levels of Mo-MLV was detected by qPCR.

Supplementary Figure 1

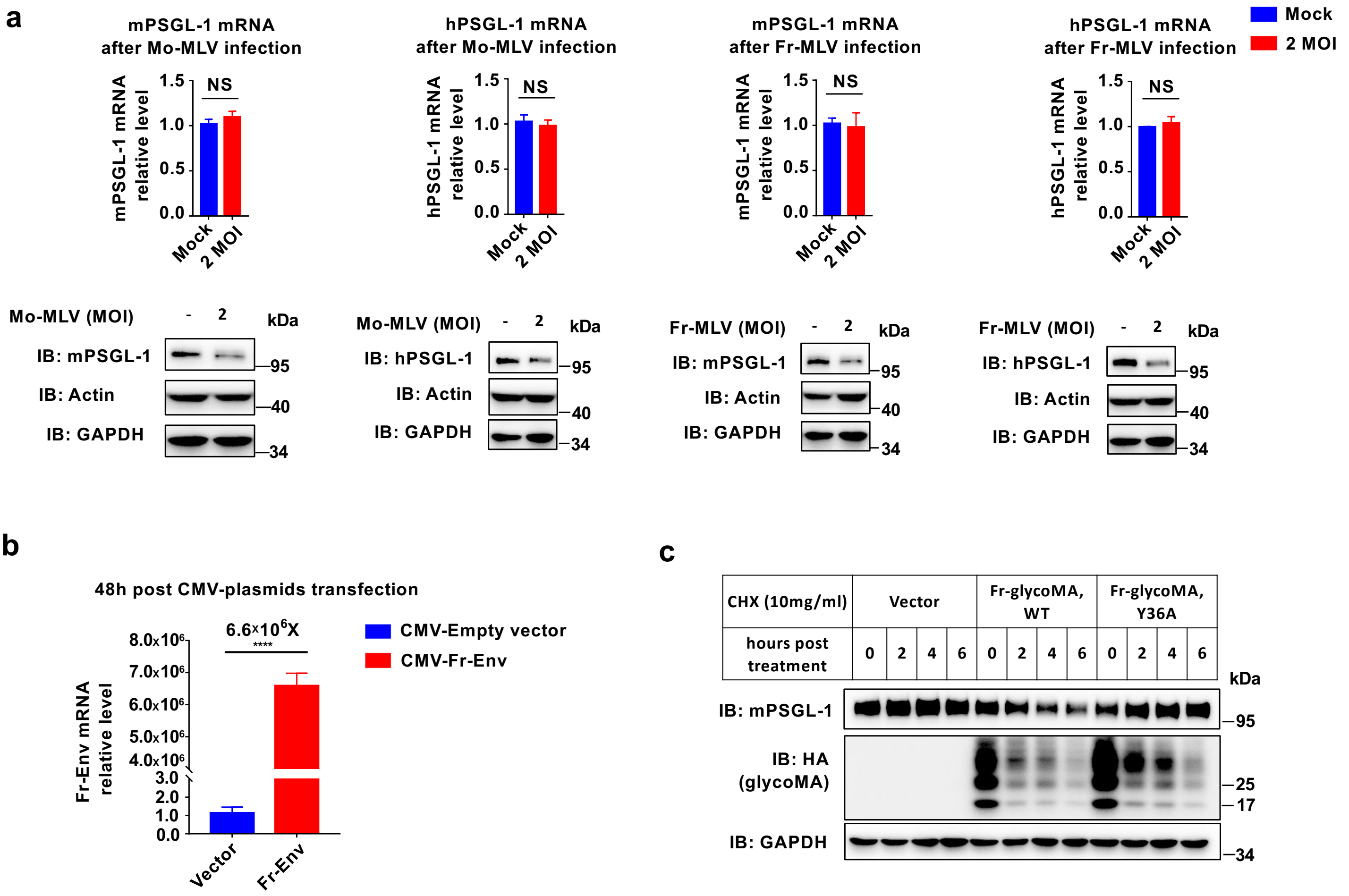

Supplementary Figure 2. a, 3T3 stable cell lines stably expressing mPSGL-1 or hPSGL-1 were infected with Mo-MLV or Fr-MLV, 48 hours post infection cells were harvested and analyzed by RT-qPCR and Western blot to detect the mRNA level or protein level change of PSGL-1. **b,** The relative mRNA level of Fr-Env in 293T cells co-transfected with mPSGL-1 plasmid and Env component of Fr-MLV plasmid were detected by RT-qPCR compared to that of cells transfected with CMV-vector plasmid post 48h plasmids transfection. **c,** Another repeat of this experiment as that of **Fig. 2e**: 293T cells were co-transfected with plasmids expressing mPSGL-1 and glycoMA, glycoMA Y36A mutant or an empty vector. One day after transfection, cells were treated with CHX and collected at the indicated time points.

Supplementary Figure 2

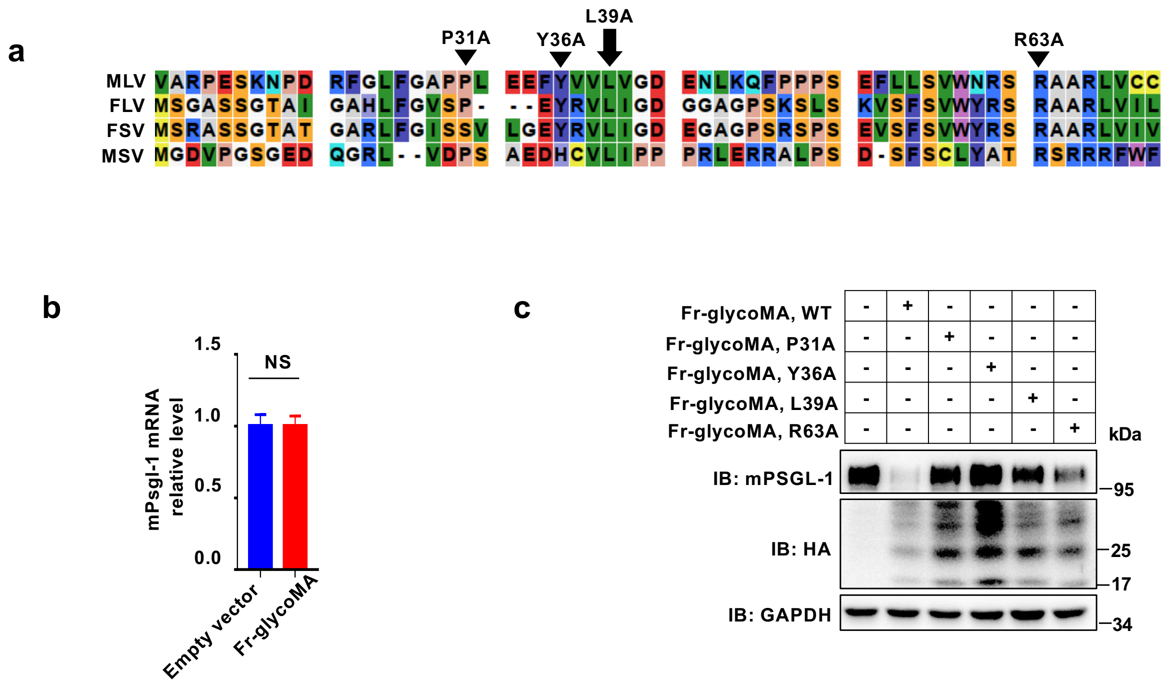

Supplementary Figure 3. **a**, The N-terminal amino acid sequences of glycoGag of MLV, feline leukemia virus (FLV), feline sarcoma virus (FSV), and Woolly monkey sarcoma virus (MSV) were aligned. Residues targeted for mutations are indicted by arrowheads. **b**, 293T cell were transfected with the plasmids of mPSGL-1 and Fr-glycoMA or an empty vector, 48hr later cell were harvested and analyzed by RT-qPCR to detect mRNA level of mPSGL-1 difference between two groups. **c**, Fr-glycoMA mutations mentioned above were co-transfected with the plasmid of mPSGL-1 into 293T cell, 48hr later cell lysates were analyzed by Western blot.

Supplementary Figure 3

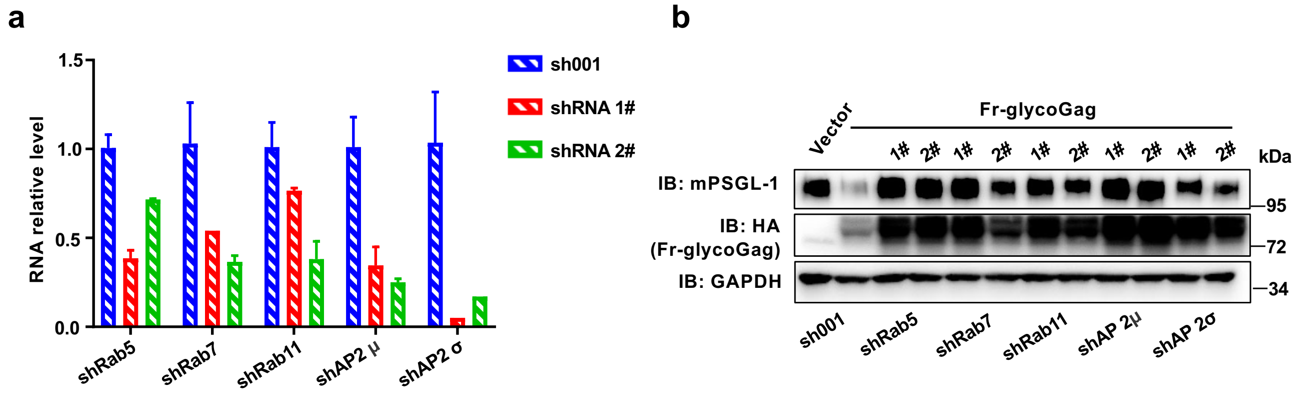

Supplementary Figure 4 a, The indicated genes are knocked down by shRNA in 293T cells, the blue column with stripe indicates the expression level of the shRNA target gene in cells stably expressing a non-targeting control shRNA, the red column with stripe indicates the expression level of the shRNA target gene in cells stably expressing the indicated shRNA. **b**, 293T cells stably expressing indicated shRNA were co-transfected with mPSGL-1 and glycoGag for 48h and analyzed by Western blotting. Empty vector was used as a negative control for the glycoGag expressing vectors.

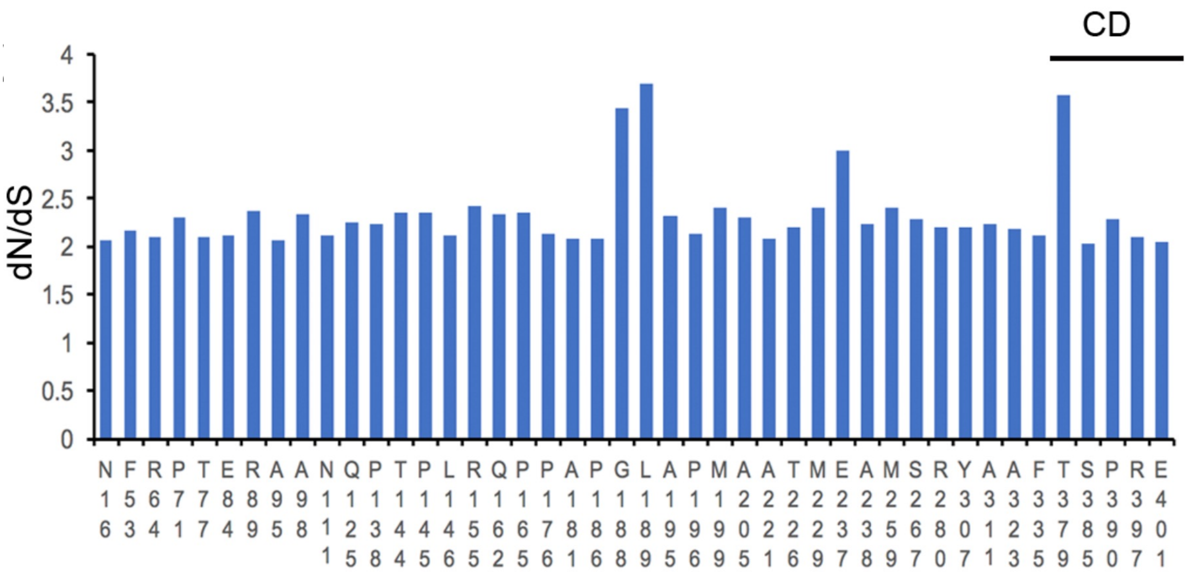

Supplementary Figure 5. dN/dS ratio of all the residues of PSGL-1 that have a dN/dS higher than 1. The ratios were calculated by PAML . CD: C-terminal domain.

Supplementary Figure 5

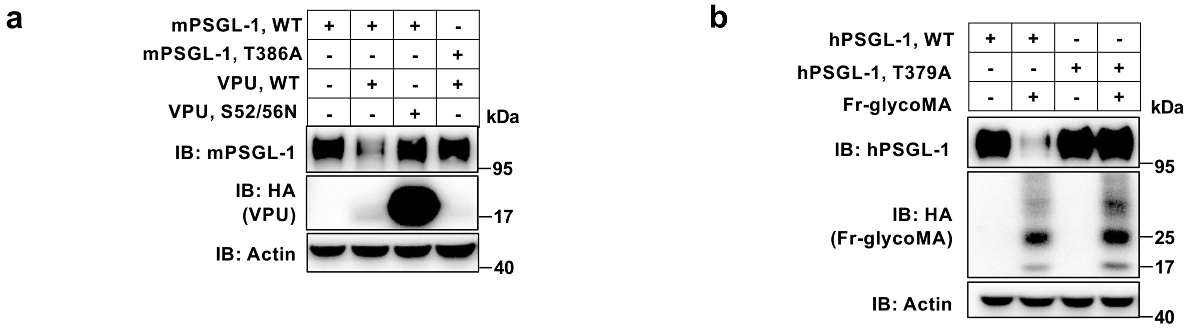

Supplementary Figure 6. a. The plasmids of wildtype and T386A mutant of mouse PSGL-1 were co-transfected with plasmids of wild type of HIV-Vpu and a loss-of-function mutant of S52/56N of Vpu as control into 293T cell, 48hr later the cell lysates were harvested and analyzed by Western blot. **b.** The plasmids of wildtype and T379A mutant of human PSGL-1 were co-transfected Fr-glycoMA expressing plasmid (+) or an empty vector (-) into 293T cell, 48hr later the cell lysates were harvested and analyzed by Western blot.

a

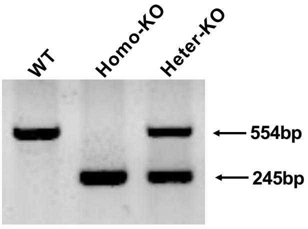

b

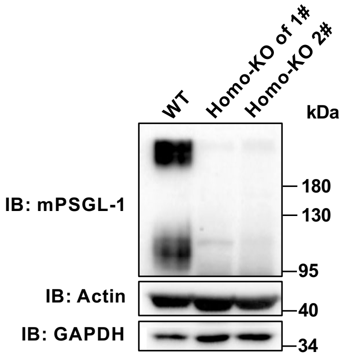

Supplementary Figure 7. a. Genotyping to identify WT, homozygote and heterozygote of mouse PSGL- KO mice. **b.** the Knockout effect of PSGL-1 were verified by the Western blot of spleen samples got from WT mice and two homozygotes littermates.

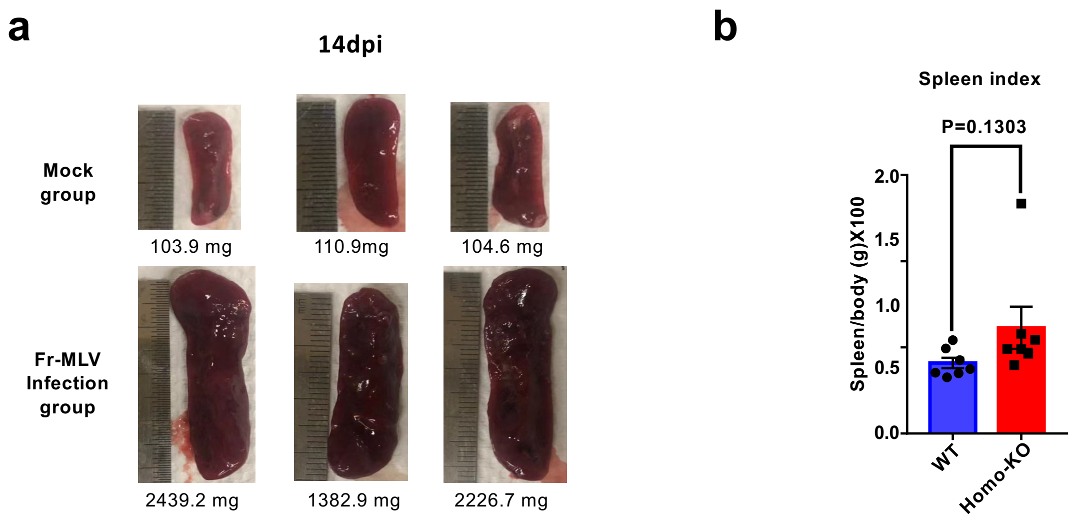

Supplementary Figure 8. a, Spleens tissues were harvested 14dpi of Fr-MLV infected with 300 MOI for passage of virus and weight of tissues were shown below each tissue. **b,** WT and KO mice were infected with 50 MOI Fr-MLV for 7 days before being sacrificed. Spleens and bodies weights of the mice were measured to calculate spleen index (Spleen/body (g)X100).

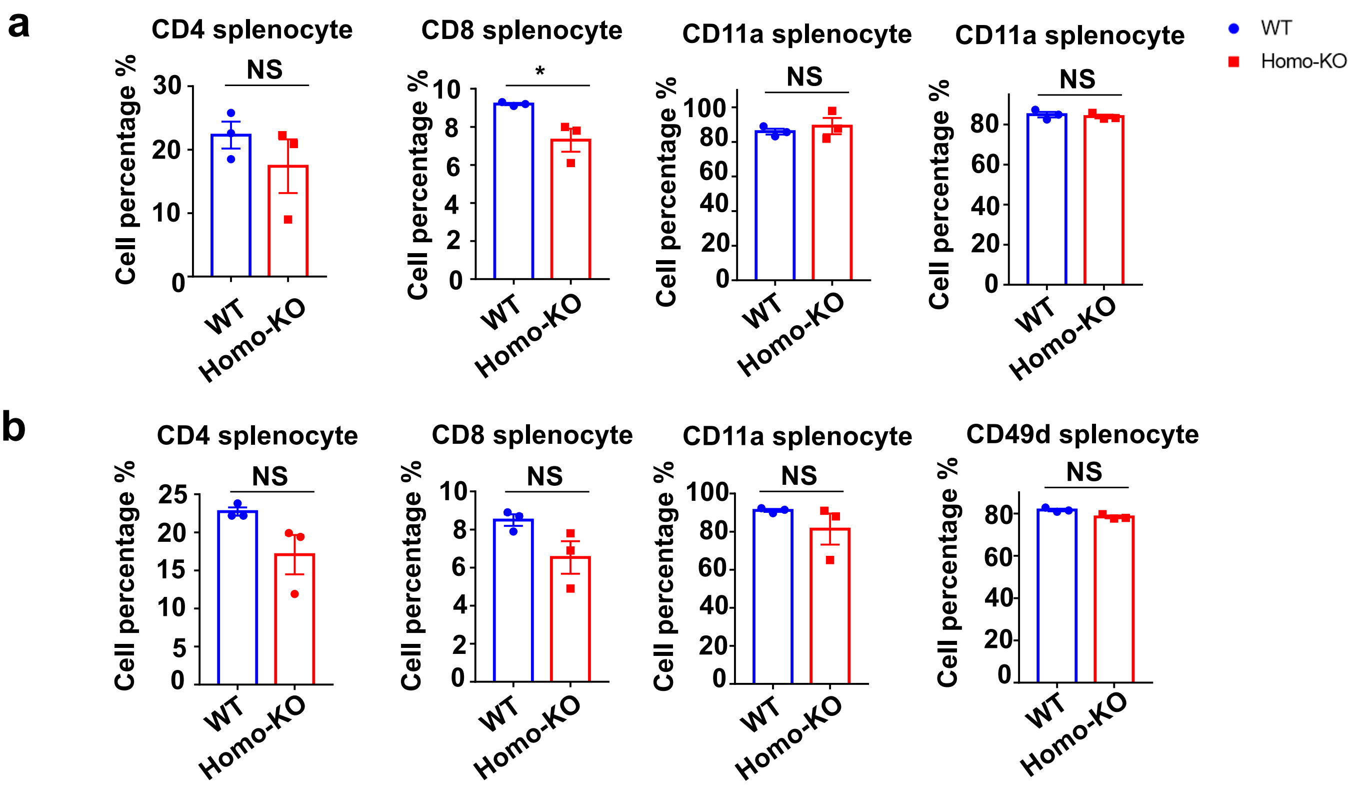

Supplementary Figure 9. a. Identify the ratio of splenocyte expressing CD4, CD8, CD11a and CD49d in WT, homozygote of mouse PSGL- KO mice by flow cytometry, which were uninfected. **b.** Identify the ratio of splenocyte expressing CD4, CD8, CD11a and CD49d in WT, homozygote of mouse PSGL- KO mice by flow cytometry, which were infected with Fr-MLV 7 days post infection.

Supplementary Figure 9
